# Neurogenic Inflammation in Allergic Contact Dermatitis

**DOI:** 10.3390/biomedicines13030656

**Published:** 2025-03-07

**Authors:** Ernesto Aitella, Massimo De Martinis, Ciro Romano, Gianluca Azzellino, Lia Ginaldi

**Affiliations:** 1 Department of Life, Health and Environmental Sciences, University of L’Aquila, 67100 L’Aquila, Italy; ernesto.aitella@graduate.univaq.it (E.A.); massimomariamarcello.demartinis@univaq.it (M.D.M.); gianluca.azzellino@aslteramo.it (G.A.); 2 Clinical Immunology Outpatient Clinic, Division of Internal Medicine, Department of Advanced Medical and Surgical Sciences, “Luigi Vanvitelli” University of Campania, 80131 Naples, Italy; ciro.romano@unicampania.it

**Keywords:** allergic contact dermatitis, neurogenic inflammation, substance P, mast cell, MRGPRX2, endocannabinoid system

## Abstract

Allergic contact dermatitis (ACD) is a skin condition characterized by inflammation resulting from hypersensitivity upon contact with certain allergens. Although ACD is characterized by an immune-mediated pathomechanism, the involvement of the nervous system in this condition has increasingly been considered, particularly in the amplification and persistence of inflammation. This paper aims to present a comprehensive overview of the mechanisms involved in neurogenic inflammation in ACD, focusing on the role of sensory neurons, the release of neuropeptides, their interaction with immune cells, and the potential therapeutic implications related to neurogenic pathways, diversified by age and gender. Innovative therapies for ACD, including topical formulations, may target the mass-bound X2 G-protein-coupled receptor (MRGPRX2) and endocannabinoid systems.

## 1. Introduction

Allergic contact dermatitis (ACD) is a common skin condition, in some cases of professional relevance. Even though it is characterized by a cell-mediated immunological mechanism and by the recruitment of lymphocytes, mast cells (MCs), and eosinophils, ACD differs from other allergic conditions where the central pathophysiological role is played by the IgE/FcεRI axis [1].

Cellular and tissue structures of the skin are involved in the associated cutaneous lesions and pruritus, together with immune cells—located within or afferent to the skin—and sensory nerve fibers. Due to their possible interaction, the skin system, therefore, can be considered an organ susceptible to neurogenic inflammation, starting from the presence of neuropeptide receptors on immune cells themselves, such as on MCs [2].

On the other hand, MCs are ancient but not yet fully discovered cells, which have demonstrated phenotypic and functional diversity even in more recent times. In particular, the identification of the mass-bound X2 G-protein-coupled receptor (MRGPRX2) on skin MCs has led to the clarification of their involvement, not only in IgE-mediated reactions but also in so-called pseudoallergy, contributing to the interesting field of neurogenic inflammation [3].

Thus, the exploration of neuroinflammation in a condition with an allergic background like ACD allows researchers to investigate histamine-independent pathophysiological mechanisms and potential therapeutic implications, in particular in severe forms and chronic or unresponsive pruritus.

## 2. Allergic Contact Dermatitis

ACD is a skin disorder caused by a type IV hypersensitivity reaction after repeated skin exposure to sensitizing substances, leading to erythema, edema, pruritus, or vesicles and blisters, typically not appearing immediately after contact [4].

This disorder represents 20% of cases of contact dermatitis. Among contact-induced dermatitis, systemic contact dermatitis, contact urticaria, and protein contact dermatitis also have to be counted; moreover, in the majority of cases, a localized nonimmunological reaction defines irritative contact dermatitis, characterized by the disruption of keratinocytes and barrier permeability, T-cell activation, and cytokine release, and, above all, intracellular adhesion molecule 1 [5].

Additionally, in ACD, after the sensitization phase, re-exposure to haptens determines their diffusion into the skin, with a haptenated peptide complex presentation to memory T lymphocytes and innate and adaptative immune responses, with the upregulation of adhesion molecules and chemokines by keratinocyte, dendritic cells, endothelial cells, and fibroblasts, as well as MCs, leading to recruitment to the involved site of T cells, NK cells, neutrophils, macrophages, and also eosinophils [6,7]. In the context of effector T lymphocytes, both CD4+ and CD8+ seem to have a role in ACD as primary and main effector cells, respectively [8], while, regarding the subphenotypes of T helpers, in addition to Th1 and Th17, Th 2 and Th 22 may also be included [9].

The expansion and recirculation of these effector cells is the result of the previous activation of dermal dendritic cells and Langerhans cells in the sensitization phase and their migration and maturation to draining lymph nodes.

The sequence of events starts with the stimulation of pathogen-associated molecular pattern (PAMP) receptors, such as Toll-like receptors (TLRs) and NOD-like receptors (NLRs), by damage-associated molecular patterns (DAMPs) and reactive oxygen species (ROS). This process is also influenced by keratinocytes, which play a role in the enzymatic transformation of prohaptens into haptens and the generation of alarmins and cytokines (Table 1).

Overall, the inflammatory process is predominantly advanced by INF-γ, TNF-α, IL-4, and IL-17, and it is characterized by histologically marked spongiosis and by aspects of a loss of cohesion, destruction, and desquamation at skin contact sites [10].

The subsequent resolution phase aims to interrupt this inflammatory process, essentially through the clearance mechanism of the hapten and, in particular, with the intervention of regulatory T cells.

Acute and chronic forms of ACD can be distinguished, with implications in the management of cutaneous lesions and pruritus; a patch test is the gold-standard diagnostic tool in ACD and nickel-induced dermatitis remains one of the most frequent forms. Other common allergens are cobalt chloride, copper sulphate, chromium, fragrances, balsam of Peru, colophony, paraphenylendiamine, formadehyde, parabens, and also acrylates, hair dyes, rubber chemicals, epoxy resins, vehicles, and antimicrobials [11,12].

ACD lesions may resemble other types of dermatitis such as atopic eczema, even if the site, extension, and causal link with contact with haptens may be indicative, as may localization to the face, eyelid, hands, and feet. Itchy blisters on erythematous skin are more typical of acute lesions, but may persist in subacute forms. Erythema and desquamation prevail in chronic forms, leading to lichenification, with a thickening of the skin and a prominence of the edges of the lesions [13]. Interestingly, several cases of lichen simplex chronicus and/or prurigo nodularis with concomitant clinically evident allergic contact sensitization, particularly to p-phenylendiamine contained in hair dye, have been reported [14]. Photosensitive lichenoid dermatitis and prurigo nodularis can infrequently be part of the clinical features associated with Parthenium dermatitis, a plant-related dermatitis triggered by the airborne allergen from the Compositae weed Parthenium hysterophorus, potentially involving a combination of immediate and delayed hypersensitivity mechanisms (type I and type IV) [15].

Therefore, the differential diagnosis of ACD may represent an important challenge in order to properly avoid the triggering allergens or the worsening of other skin conditions that may mimic ACD, such as dyshidrotic eczema, asteatotic eczema, seborrheic dermatitis, nummular eczema, stasis dermatitis, delayed-type hypersensitivity drug reactions, psoriasis, and skin infections [16].

We now introduce the concept of neurogenic inflammation and discuss its contribution to ACD.

## 3. Neurogenic Inflammation, Allergy, and Skin

The connection between neurogenic inflammation and allergy has been known at least since the last century. In its original definition, the plasma leakage in the postcapillary venules of skin and airways is highlighted in response to the peripheral sensory nerve stimulation [17].

In particular, substance P (SP) is considered the primary neuropeptide mediator released from capsaicine-sensitive unmyelinated sensory nerve fibers and able to act via tachykinin Neurokinin 1 (NK-1) receptors. Interestingly, SP may induce transient focal and reversible gaps in the intercellular junctions of endothelial cells. Among tachykinins, neurokinin A can also lead to vasodilatation, plasma exudation, bronchoconstriction, and mucus secretion in airways. Moreover, in asthma, the damaged epithelium exposes sensory nerves to inflammatory products and increase neuropeptide release as well as bradykinin, contributing to the maintenance and amplification of local inflammation, while the loss of endopeptidases from the epithelial cells cannot contrast at all with the further effects of tachykinins [18].

It is no coincidence that, among drugs used in asthma, GINA guidelines recommend anticholinergics [19].

In the skin, the neurogenic inflammatory pathway is composed of keratinocytes, melanocytes, fibroblast, MCs, Langherans cells, or endothelial cells, that are in communication with peripheral nerve endings via neuroptophins and neuropeptides. The further release of neuromediators exacerbates the skin inflammation involving, in particular, MCs, Ca^2+^-dependent ion channels, TH2CD4+ cells, and their cytokine production of IL-4, IL-13, and IL-31 [2].

We already speculated about the possible neurogenic interconnections between cutaneous disturbances, pseudoallergy, and Th2-like profile pathomechanisms, such as in drug [20] and food [21,22] adverse reactions and in the overlap syndrome of urticaria and gastroesophageal reflux disease [23].

Neurogenic inflammation in the skin represents a physiological procedure to maintain homeostasis versus chemical, physical, and biological signals, starting from the terminals of afferent unmyelinated C-fibers and myelin-type delta A-fibers; in the late nineteenth century, the experimental stimulation of dorsal roots confirmed how SP and calcitonin gene-associated peptide (CGRP) released via nociceptors induce vasodilation and the permeability of blood vessels with a supposed direct mechanism on vascular endothelial cells [24].

SP and CGRP are also powerful histamine releasers from MCs, which contribute to plasma leakage in the skin and amplify this neuroimmune cross-talk.

Similarly to peripheral events, neurogenic inflammation can persist at the central level through a mechanism of central sensitization up to phenotypic modifications of the nerve fibers due to the ongoing alterations in neuroexcitability [25]. Allodynia and alloknesis may represent clinical manifestations of this conditions in chronic dermatitis and atopic dermatitis (AD) [26,27].

Moreover, evidence of neurogenic inflammation in the skin may be proven by the increased expression of neurotrophins and peptidergic nerve fibers in chronic conditions, such as AD, rosacea, psoriasis, and chronic idiopathic urticaria [28,29,30].

However, “neurodermatitis” features have been stressed among the criteria to validate mouse models of AD and also show some evidence in ACD [31].

## 4. Evidence of Neurogenic Inflammation in ACD

Regarding mouse models, Botz et al. explored the neuroinflammatory role of galanin in oxazolone-induced contact dermatitis. Galanin is a neuropeptide widely represented in the central and peripheral nervous system, with a demonstrated role in murine contact allergy [32], and, in particular, in inhibiting neurogenic edema through a GAL 3-dependent mechanism [33]. Anyway, in this study, GAL-3-receptor-deficient mice did not show significant variations after the oxazolone challenge compared to the wild-type mice. Thus, even if a correlation with GAL 3 seems to be excluded in the oxazolone-induced ACD, it retains its own anti-edema role, limited to the pathophysiological mechanisms associated with neurogenic inflammation and not in the immunological pathways.

Differently, acute stress in the absence of previous sensitization, through SP, NK-1 receptor, and nerve growth factor, may induce neurogenic skin inflammation in mice [34]; moreover, the activation from the stress of a homologous hypothalamic–pituitary–adrenal (HPA) axis in the skin determines the exacerbation of many dermatological diseases through the release of proinflammatory hormones, neuropeptides, and neurotrophins, in particular, through MCs’ stimulation by corticotropin-releasing factor (CRF) [2,35,36,37].

Conversely, Liu et al. identified several differentially expressed genes related to skin neuroinflammation, itch, and pain in an ACD model using transcriptomic RNA sequencing. Among the Mas-related G-protein-coupled receptors (Mrgprs), they found an increased expression of MrgprD in dorsal root ganglia neurons that innervate the inflamed skin, suggesting it as a potential therapeutic target [38].

## 5. Mast Cells: Neuroimmune Interactions

MCs are bone-marrow-derived and tissue-resident immune cells, typically situated as gatekeepers at the interface of the host and the environment, in particular, around blood vessels and nerves [39].

First described by Paul Ehrlich in 1878 as cytoplasmic granulated cells and known for their metachromasia, they are enriched in heparin and characteristically contain the proteases tryptase and chymase [40].

Among granules, preformed, neoformed, and neosynthesized mediators related to their early, late, and chronic phases of actions and function can be distinguished, including tissue remodeling and fibrosis. In particular, other preformed mediators are represented by the proteases Carboxypeptidase A, Granzyme B, and Matrix metalloproteinases, lysosomal enzymes, chemokines, cytokines, SP, Vasoactive Intestinal Peptide, and Eosinophil Major Basic Protein. Furthermore, the neoformed phospholipid metabolites eicosanoids, prostaglandins D2 and E2, and leukotrienes B4 and C4 determine the recruitment of immune effector cells, permeability of blood vessels, contraction of smooth muscle, production of mucus, or stimulation of sensory neurons [41,42,43,44].

In humans, at least six distinct transcriptomic clusters of MCs have been discovered across 12 different organs, indicating their presence in connective tissue or sub-epithelial areas of the respiratory mucosa, gastrointestinal tract, heart, and skin, where they function as resident long-lived cells. In these locations, N-cadherin or synaptic cell adhesion molecules characterize the synaptic-like connections established between MCs and neurons [45]. Mature human MCs can be categorized into two types: MCTC, which contains tryptase, chymase, carboxypeptidase, and cathepsin, primarily located in the skin, and MCT, which expresses only tryptase and is found in the lung and in the gut [46]. MCTCs are typically found within the dermis and also near the nerve endings in the epidermis, and they have been identified in cases of psoriasis [47].

Surface receptors include high/low-affinity FcεRI and FcεRII receptors, the FcγRIIA receptor; the KIT receptor with an affinity for the stem cell factor; and G-protein-coupled receptors (GPCRs); as well as the complement component C3a receptor, cannabinoid receptor type 1 and 2 (CB1 and CB2), histamine receptors type 1 and 4 (H1R, H4R), and MRGPRX2 [48].

In Type 1 hypersensitivity, as described by Gell and Coombs, IgE antibodies bind to the high-affinity receptor FcεRI on MCs and basophils that have been previously sensitized. Upon subsequent exposure to multivalent allergenic antigens, these cells degranulate and activate due to crosslinking; the immediate effects that follow include vasodilation, bronchoconstriction, and allergic inflammation, which are characteristic of conditions such as allergic rhinitis and asthma, food allergies, and allergic responses to venom or medications, as well as IgE-mediated types of urticaria-angiedema and anaphylaxis [49].

MC degranulation independently from the IgE/FceRI axis is described for some types of pathogen-mediated MC activations, and physical stimuli, as well physical urticaria, part of the adverse reactions to contrast media or cationic groups of neuromuscular blocking agents, morphin, fluroquinolone and vancomycin, autosominal dominant vibratory urticaria, hereditary alfa-tryptasemia, mastocyotosis, and the MC activation syndrome [50,51,52,53,54]. Several receptors are involved in these pathways, such as TLR, NLRs, RIG-I-like receptors (RLRs), and FcγRI-III/complementary receptors, the adhesion G-protein-coupled receptor E2 (ADGRE2 or EMR2), or the mutant ion channel TRPV2 for mechanical, thermal, and osmotic stimuli, NOX2 in response to UVA irradiation, the suppression of tumorigenicity 2 (ST2), and P2X1 receptors for alarmins. However, the deepening of non-IgE-mediated pathways received a significant boost by the re-evaluation of MRGPX2 after the first demonstration of its expression in MCs in 2006 by Tatemoto et al. [55].

In MCs purified from human skin, MRGPRX2 activation by the agonist compound 40/80 and SP showed that it requires Gαi and Gαq, the crucial role of Ca++ channels and PI3K, and the additional participation of ERK1/2. The MRGPRX2-mediated degranulation is characterized by a rapidness of response (within around 1 min with respect to the time-course of FcεRI aggregation-related events) [56]. Moreover, in drug-naïve patients, MRGPRX2 seems to correlate, above all, with mild-to-moderate adverse reactions, particularly in the presence of eosinophil/basophil-driven comorbidities or in chronic urticaria, where MRCGPRX2-positive cells result increased [57].

Interestingly, MRGPRX2 is found predominantly in cutaneous MC, but, recently, its expression has been described also in eosinophils and basophils [58,59].

In AD, both the number of MCs and expression of SP and PAR2 are increased, whereas genes encoding MRGPRX2 are upregulated. In particular, Staphylococcus Aureus may induce MC degranulation via MRGPRX2 by δ-toxin or Human β-defensin 2 from keratinocytes [60], while Thymic Stromal Lymphopoietin (TSLP) has been shown to degranulate MCs through MRGPRX2 in a STAT5-dependent and JNK-supported manner [61].

Moreover, in rosacea, the overexpressed host defense peptide LL-37 contributes to the aberrant activation and degranulation of cutaneous MCs via MRCGPRX2, leading to the typical erythema, flushing, burning sensation, and itching [62,63].

Therefore, MCs exhibit heterogeneity and plasticity as they can be driven by the cytokine milieu, sensor molecules, and cell surface receptors, along with the capacity to “transdifferentiate” from one phenotype to another [64,65]. Moreover, according to a new and more dynamic vision, MCs are so involved not only in the typical IgE-mediated hypersensitivity, but only in pseudoallergy and neurogenic inflammation.

Although “-omic” studies are changing the pathogenetic, diagnostic, and therapeutic approach to allergic skin diseases, current evidence of these pathways in ACD still has a limited role [66,67]. Furthermore, most of the considerations reported in the literature mainly refer to type I hypersensitivity mechanisms, that definitely do not find a place in ACD. However, the role of the MCs at the crossroads of hypersensitivity reactions and neurogenic inflammation should not be overlooked in neuroimmune interactions and these limitations offer the opportunity to carry out further studies and gain new perspectives in ACD [68].

## 6. MRGPRX2: Potential Antagonists?

Mast cells, mostly distributed in the skin and in intimate synaptic-like contact with sensory peptidergic nerves [69,70], seem to have a central role in neurogenic inflammation and act as a key modulator in allergic diseases [71].

Pivotal evidence of neuroimmune inflammation and MC contribution is the increased number of MCs in skin lesions from patients with ACD and, on the other side, the higher expression of pro-adrenomedullin peptide 12 (PAMP12), a ligand of MRGPRX2, compared to healthy subjects: experiments on MrgprB2^MUT^ mice highlighted differences in scratching, itch behavior, and immune cell recruitment, suggesting the involvement of IgE/FcεRI-independent pruritogenic pathways [72].

In particular, PAMP12 seems to stimulate the release of tryptase β2 more than histamine and serotonin. In the skin, tryptase binds to the protease-activated receptor 2 (PAR-2 receptor), releasing neuropeptides such as SP and CGRP, which are responsible for itching, scratching, or pain perception [2]. Thus, the consequent interaction of tryptase β2 with protease activated receptors on sensory neurons may lead to pruritogenic stimulation in a histamine-independent manner [73].

Moreover, SP elicits inflammatory responses via MRGPRX2, involved in non-histaminergic itch, together with cationic peptides released from eosinophils, neutrophils, and epithelial cells.

The potential limits of an anti-histamine modulation across this neuronal axis may have their basis in this neuro-immune interaction [60].

In this scenario, finding any antagonist of MRGPRX2 to modulate MC degranulation and neuroflogistic-related aspects could be interesting: in the literature, several potential antagonists have been reported, including isoflavones and flavonols such as genistein and quercetin, isoliquiritigenin from licorice, piperine from black pepper, and shikonin, a naphthoquinone from Chinese herbal medicine [74].

Suzuki et al. investigated an antagonistic DNA aptamer that targets MRGPRX2 in rats, leading to a decrease in histamine release, suggesting a potential relevance in perioperative anaphylaxis [75].

In the context of the development of new molecules [76,77], notably, Wollam et al. have recently discovered small-molecule antagonists of MRGPRX2 that inhibit the degranulation of MCs in vitro, in vivo, and ex vivo within human skin [78].

## 7. Endocannabinoid System and Topical Pharmacologic Implication

**Figure 1:**
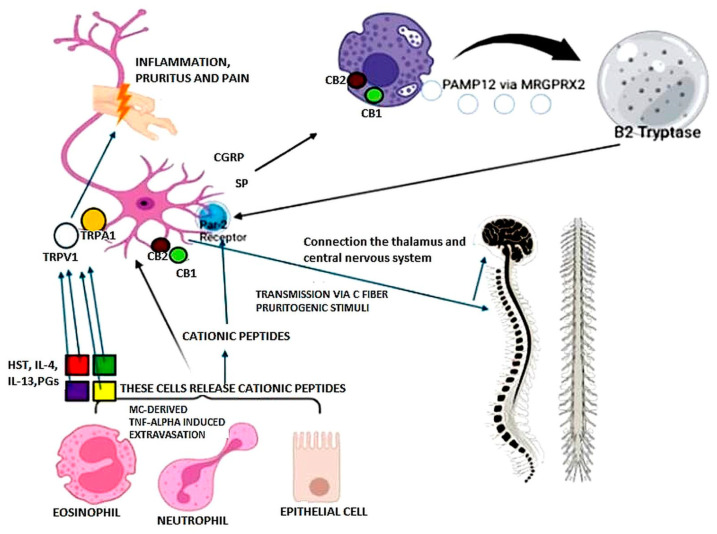
Neurogenic contribution to allergic contact dermatitis.

After the activation of pruritogenic receptors, exogenous and endogenous pruritogen stimuli are conveyed through unmyelinated C fibers from the epidermis and dermal districts to the contralateral spinothalamic tract, thalamus, and cortex (Figure 1). Cannabinoid receptor 1 and 2 (CB1 and CB2) are G-protein-coupled receptors involved in neurogenic and inflammatory pain and itch, and their agonism showed antipruritic effects at both the central and peripheral levels, in systemic and dermatologic diseases, as well as AD and ACD. Although the modulation of CB1 is more effective in the central nervous system, CB1 and CB2 are both found on the fibers of peripheral cutaneous nerves and in MCs, and they might affect the recruitment of immune cells and skin inflammation in dermatitis models [79,80].

In skin lesions of ACD, in addition to histamine (HST), proinflammatory cytokines, and prostaglandins (PGs), the highly expressed pro-adrenomedullin peptide 12 (PAMP12) stimulates, via Mas-related G-protein-coupled receptor member X2 (MRGPRX2), the augmented MCs to release tryptase β2. After its binding with the protease activated receptor 2 (PAR-2 receptor) on sensory neurons, substance P (SP) and gene-related peptide CGRP are released. Furthermore, SP elicits inflammatory responses via MRGPRX2 together with cationic peptides released from eosinophils, neutrophils, and epithelial cells, leading to itching, scratching, and pain perception, in an IgE/FcεRI- and histamine-independent manner. On the other hand, the activation cannabinoid receptor 1 and 2 (CB1 and CB2) and transient receptor potential ion channels (TRPV1, TRPA1) on sensory neurons convey the exogenous and endogenous pruritogen stimuli to the contralateral spinothalamic tract, thalamus, and cortex, through unmyelinated C fibers. MCs also express both CB1 and CB2 receptors, while they induce neutrophil extravasation by tumor necrosis factor-α (TNF-α) production.

The transient receptor potential (TRP) are ion channels that activate cutaneous nerve fibers in sensory responses. TRP vanilloid 1 (TRPV1) and TRP ankyrin 1 (TRPA1) have a role in neurogenic inflammation and itch sensation and can interact with cannabinoids [81]. In particular, palmitoylethanolamine (PEA) and cannabidiol (CBD) antagonize and desensitize TPRV1 with anti-pruritic effects and without direct interactions with CB1 and CB2. Moreover, 2-arachidonoylglycerol, anandamide, and Δ (9)-tetrahydrocannabinol can interact with CB1, CB2, and TRPV1 to alleviate pruritus.

Interestingly, the lipophilic properties of cannabinoids can be directly exploited on the skin through topical transdermal applications, in local limited areas affected by ACD, with a prevalent interest in non-THC cannabinoids, as well as PEA and CBD [79].

Clinical trials involving PEA or the topical treatment analog adelmidrol have demonstrated advantages in managing atopic dermatitis (AD), asteatotic eczema, uremic itch, and/or chronic itching [82,83,84], underlining the histamine-independent component in the physiopathology under these conditions and their possible refractoriness to conventional anti-histamine therapies [85].

Instead, an increase in pruritus may be found in the contest of allergic reactions to cannabinoids [86].

## 8. Age and Gender Influences on ACD and Neurogenic Inflammation

ACD is conditioned by several factors, including sex and age. Skin aging and immunosenescence can modify the features of ACD, and a female predominance has been observed in patients over 60 years old [87]. Furthermore, the impact of sex and age on ACD is also mediated by hormonal influences. Neurotransmitters, endocrine factors, and cytokines released from nerve endings act on specific receptors in the skin and communicate among themselves. Dysfunctions in these systems lead to cutaneous inflammatory pathologies, such as autoimmune and allergic diseases, skin aging, and cutaneous malignancies. The skin can, therefore, be considered a neuroendocrine organ.

The compromised barrier function in aged subjects and the age-related microbiota remodeling are contributing factors for both allergen penetration and sensitization [88]. The microbiota on the cutaneous surface, through its metabolic activity, modulates the immune and neuroendocrine activity of the skin. The age-related impairment of the skin barrier leads to the easier penetration of allergens, and changes in the microbiota during aging facilitate sensitization [89]. Interactions between the nervous system, immune functions, and microbiota are topics of translational interest in the field of intestinal and skin diseases [90]. Metabolic, genetic, and hormonal influences also contribute to skin damage and modulate the onset and manifestations of ACD.

Studies on the correlations between contact allergic sensitivities and age in ACD are not univocally concordant as they are conditioned by the study design, population, genetic and geographic factors, and environmental exposure [91]. However, it would seem that older people have, more frequently, multiple contact allergies compared to younger people, probably due to the greater potential time of exposure to allergens, the greater use of topical drugs, and the age-related skin barrier alterations shown. Although, in some trends, the prevalence of sensitization increases progressively with age, if pediatric populations are included, the overall incidence is higher in children up to 10 years. The peak ages for positive reactions to patch tests may also be earlier in women than in men. With reference to the main haptens, sensitization to nickel and cobalt would be greater in individuals < 30 years than in those > 50 years, in particular, among women, for nickel. Positive reactions to balsam of Peru would be more frequent in men over 40 years. Instead, allergy to fragrances prevails in women and increases with age. These data have to be further investigated but they are influenced by the gender-dependent exposure to haptens, by immunoendocrine factors linking sex hormones and the immune system, and by immunosenescence. The neuro-endocrine network, which plays important roles in the regulation of skin immunity, is altered during aging. Age-related Vitamin D deficiency is associated with increased skin inflammation [92]. Vitamin D3 regulates the hypothalamic–pituitary–adrenal axis (HPA). In particular, 1,25(OH)2D3 stimulates the expression of neuropeptides and their receptors in keratinocytes [93].

Gender differences in skin structure and physiology, as well as in allergen exposure, also influence ACD onset and development [94]. For example, postmenopausal women present changes in skin trophism related to the decrease in estrogen that make them more vulnerable to the onset of ACD [95]. In addition, the neurogenic component is also influenced by age and gender. Stress impacts the skin directly (via the peripheral nervous system) or indirectly (via the immune and endocrine systems). Inflammaging and hormonal influences both impact the neurogenic component of ACD, i.e., the participation of nerves and their mediators (neuropeptides) to the inflammatory processes of ACD [18]. TRPV1 expression is higher in aged skin, suggesting that TRPV1 may be related to senile pruritus and increased neurogenic inflammation in the elderly [96]. Sexual dimorphism exists in neuroimmune communication pathways. Estrogen and progesterone impact the release of neurogenic infammatory neuropeptides, such as SP and CGRP [97].

Several neurogenic factors exacerbate skin inflammation in ACD differently in males and females. Opioids potentiate ACD more in females than in males. Sex differences in SP signaling pathways contribute to different inflammatory outcomes in females and males. For example, constitutive sex differences characterize the NK1 receptor system in the skin, through which SP acts [98]. Sexual dimorphism in TRPV-1-receptor-mediated mechanisms and greater inflammatory responses to capsaicin in females have also been demonstrated [99]. The clinical benefits of the IgE blockade with Omalizumab in itch-associated skin conditions [100] may show sexual dimorphism. The characterization of gender differences in neuroimmune crosstalk may, therefore, have potential implications for a gender-oriented clinical approach to ACD, as well as other allergic diseases with a neurogenic component [101,102].

Ultimately, several pleiotropic bioregulatory factors and hormonal signals, such as cytokines, active forms of vitamin D [93], and neuropeptides [103] released by keratinocytes, melanocytes, immune cells, and sensory nerve endings into the skin, exert a role in the complex scenario of the neurogenic inflammation of skin pathologies, including ACD. All these components could be variably modulated by sex and aging [104], as well as under physiological and pathological conditions [105,106].

## 9. Treatment and Perspectives

Definitely, ACD is a common, heterogeneous condition with multiple etiologies and morphologies. It is a problem that can affect quality of life, due to its characteristics of contact site extension, recurrence, and severity, especially in chronic forms, when it represents a professional condition and when the allergen cannot be completely avoided.

For these reasons, it is clear that research should focus on identifying alternative therapeutic strategies that can also modulate non-canonical pathways, such as those related to neurogenic inflammation, which we have addressed in this work. Innovative therapeutic options in this direction would be particularly useful in forms where conventional therapies are not fully effective or when histamine-independent mechanisms prevail in the disease’s progression.

With particular reference to pruritus, although it is a subjective symptom, it can significantly influence the progression of ACD, due to the associated scratching behaviors, implications on the severity and chronicity of the typical lesions, and superinfection at the contact sites. We have also seen how non-histaminergic pruritus, in particular, can be part of dermatological diseases, like AD and ACD, or systemic conditions, characterized by a poor response to antihistamine therapy, even when it is the dominant feature of the clinical presentation [107].

Regarding notoriously more effective therapies, such as biologic treatments in skin syndromes with an allergic component and/or the type 2 inflammatory subset, there are currently no biologic drugs specifically indicated for ACD. However, the literature reports some data about the use of biologics in particular cases of recalcitrant ACD, such as Omalizumab, Dupilumab, TNF-α inhibitors, Sekukinumab, Ustekinumab, and Rituximab [108].

These considerations highlight the need to expand the pharmacological armamentarium with advanced or innovative therapies, which could include those aimed at modulating neuroinflammation.

Currently, primary, secondary, and tertiary prevention strategies represent the cornerstone of ACD management in terms of effectiveness [109]. Among topical treatments, certainly, topical corticosteroids are available in the acute phase and should be preferred instead of oral corticosteroids. Topical calcineurin inhibitors, such as tacrolimus or phosphodiesterase 4 inhibitors, are also available, as well as the important role of barrier creams, emollients, and moisturizers. Systemic therapies or other potential therapeutic targets of interest are summarized in Table 2.

The updates about the pathogenesis of ACD highlight that, due the underlying mechanism of neuroinflammation, skin and allergic diseases should be reviewed from this point of view; moreover, neurogenic inflammation cannot act or be thought of independently, but usually plays together with the other most well-known pathomechanisms, or prevails in specific phases or conditions of the diseases, with implications on the therapeutic response. Moreover, the skin features shown above may altogether define the skin as a complex “neuroimmunoendocrine” organ [2] and these neurogenic pathways can contribute to the persistence of inflammation, as the neuropeptide release may continue also in the absence of further stimuli [25,110].

Neurogenic inflammation can be suppressed by preventing the formation of endothelial gaps through the use of anti-inflammatory medications, such as steroids. Additionally, it can be managed by inhibiting the activation of peripheral sensory nerves in the skin, either by targeting the release of neuropeptides or by blocking their receptors.

The modulation of the MRGPX2 receptor would represent a promising therapeutic strategy to target such a key mediator of neuroimmunoinflammation. Despite progress and attempts to identify new molecules inhibiting MRGPX2, to our knowledge, there are currently no clinical trials in humans. Moreover, the differences between MRGPRX2 and the rodent ortholog MrgprB2 make research even more challenging, requiring more complex study models for preclinical studies [74].

For the first group of MRGPRX2 antagonist substances listed above, mostly of botanical origin, there are insufficient pharmacodynamic and pharmacokinetic data, as well as information on safety and efficacy. However, while their systemic use might still seem far away, the pharmaceutical industry could more easily test topical formulations based on these active ingredients.

In fact, a feature that should not be overlooked in the management of ACD is that it involves an external organ like the skin, limited to areas inflamed by contact with a given hapten. This makes topical therapy, in the form of creams, lotions, emollients, oils, and patches, the preferred strategy, as it can act directly and locally on the target organ, with a generally more limited extent compared to other eczematous forms such as AD, thus ensuring potentially higher therapeutic compliance from the patient, and also in terms of product quantity and costs. By avoiding the hepatic first-pass effect, it is also possible to reduce the risk of toxic and side effects.

Regarding topical treatments, acting on ACD inflammation with cannabinoid-based topical products may be interesting and useful: we have highlighted the implications of this pathway and the possibility of avoiding psychoactive effects by using non-THC cannabinoids such as CBD and PEA; moreover, unlike the previous substances discussed, there are already preliminary clinical trials in this field, although further controlled trials are needed to confirm their efficacy. Interestingly, new alternative cutaneous treatments could help to contrast side effects due to the long-term use of topical corticosteroids, including possible irritant reactions to non-immunosuppressive barrier creams [111].

Similarly, other receptors that can be targeted include TRPV1, TRPV2, NK1, and CGRP receptors, which have shown therapeutic potential in experimental pain models [112].

Finally, the identification of biomarkers in the various neuroinflammation pathways could help to guide future research and therapeutic choices.

**Table 2:** Treatment options and perspectives in ACD.

Category	Treatment Options	Perspectives
Prevention	Primary, secondary, and tertiary prevention Hapten avoidance (not always possible)	
Topical	Corticosteroids Calcineurin inhibitors (tacrolimus, phosphodiesterase 4 inhibitors) Barrier creams Emollients Moisturizers	Antioxidants chelators [113] (#)
Systemic	Oral corticosteroids Antihistamines Cyclosporine Azathioprine Methotrexate	
Photochemotherapy	Psoralen and UVA	
Biological therapies	(Literature data, clinical reports)	Omalizumab Dupilumab TNF-α inhibitors Ustekinumab Rituximab
Non-histaminergic itch	(#Topical treatments clinical trials)	(non-THC) cannabinoid-based topical treatments CBD, PEA Interaction with CB1, CB2, TRPV1
Neurogenic inflammation	(Experimental models)	Small molecule MRGPRX2 antagonists
	(Experimental pain models)	Target therapies vs. TRPV1, TRPV2, NK1, CGRP receptors
	(No clinical trials)	Genistein Quercetin Isoliquiritigenin Piperine Shikonin Biomarkers, genomic, proteomic

## 10. Conclusions

Neurogenic inflammation demonstrated a role in the pathogenesis of ACD, contributing to the onset, amplification, maintenance, and chronicity of inflammation. The interaction between cutaneous peripheral nerves, neuropeptides, and immune cells represents a key feature of the neuroinflammatory cascade in ACD. In particular, MCs have shown a central role in neuroimmune cross-talk across allergic and neurogenic inflammation.

Targeting neurogenic pathways, above all, those related to MRGPRX2, tachykinin receptors, and endocannabinoid systems, may offer potential innovative therapies, including topical formulations, particularly in patients with refractory or chronic forms of the disease. Further research is needed to better understand the precise mechanisms underlying neurogenic inflammation in ACD and to translate these findings into effective clinical therapies.

## Figures and Tables

**Table 1:** Main cells and neuroimmune interactions in ACD.

	Main Cells or Pathways
Sensitization phase	ROS/DAMPs→TLR/NLRs KCs: enzymes (pro-hapten→hapten), alarmins, cytokines Dermal DC and LC activation, migration and maturation to draining lymph nodes Effector and memory T-cell expansion and recirculation
Elicitation phase	Adhesion molecules, chemokines, cytokines, cells recruitment: DC, KC, endothelial cells, fibroblasts, macrophages, eosinophils T cells, NK cells Mast cell→TNF-α→neutrophil extravasation CD4+ and CD8+ T cells Th1, Th2, Th17, Th22 phenotypes
Resolution phase	Regulatory T cells
	Clearance of hapten
Neurogenic contribution	Cutaneous sensory peptidergic nerves
	PAMP12
	Mast cells via MRGPRX2
	Tryptase >> histamine, serotonin
	PAR-2
	SP, CGRP
	Endocannabinoid system

ROS: reactive oxygen species; DAMPs: damage-associated molecular patterns; TLRs: Toll-like receptors; NLRs: NOD-like receptors; DCs: dendritic cells; LCs: Langerhans cells; KCs: keratinocytes; PAMP12: pro-adrenomedullin peptide 12; MRGPRX2: mass-bound X2 G-protein-coupled receptor; PAR-2 receptor: protease-activated receptor 2; SP: substance P; CGRP: calcitonin gene-related peptide.

## Data Availability

Not applicable.
